# FOXM1 mediates resistance to docetaxel in gastric cancer *via* up-regulating Stathmin

**DOI:** 10.1111/jcmm.12216

**Published:** 2014-03-14

**Authors:** Xiaoxiao Li, Ruyong Yao, Lu Yue, Wensheng Qiu, Weiwei Qi, Shihai Liu, Yasai Yao, Jun Liang

**Affiliations:** a Department of Oncology of the Affiliated Hospital of Medical College Qingdao UniversityQingdao, China; b Central Laboratory of the Affiliated Hospital of Medical College Qingdao UniversityQingdao, China

**Keywords:** FOXM1, docetaxel resistance, microtubule dynamics, Stathmin, MCAK, gastric cancer

## Abstract

Docetaxel is commonly used as an effective chemotherapeutic drug for gastric cancer patients recently. With the increasing emergence of docetaxel resistance nowadays, identification of suitable biomarkers for predicting chemosensitivity to docetaxel may be a key role for improving therapeutic effects for gastric cancer patients. In this study, we investigated the correlation between the expression of transcription factor forkhead box protein M1 (FOXM1) and chemotherapy response to docetaxel in gastric cancer, the possible mechanism for which was further explored. As a result, FOXM1 overexpression was shown to mediate resistance to docetaxel in gastric cancers. It altered microtubule dynamics to protect tumour cells from docetaxel-induced apoptosis. Mechanistic investigations revealed that tubulin-destabilizing protein Stathmin, which mediated docetaxel resistance in FOXM1-silenced gastric cancer cells, is a direct down-stream target of FOXM1, whereas another microtubule dynamics protein mitotic centromere–associated kinesin (MCAK), shown to be related to docetaxel resistance in gastric cancer cells, is not associated with FOXM1 expression significantly. These results were further provided by immunohistochemical analysis, indicating that FOXM1 and Stathmin expression levels were correlated in 103 post-operational gastric cancer specimens. Moreover, when we attenuated FOXM1 expression with FOXM1 inhibitor thiostrepton, docetaxel resistance in gastric cancers was found to be reversed, simultaneously with the down-regulation of FOXM1 and Stathmin. Therefore, FOXM1 can be a useful marker for predicting and monitoring docetaxel response. Through the inhibition of FOXM1, docetaxel resistance can be reversed, and thus FOXM1 could be a new therapeutic target in docetaxel-resistant gastric cancer.

## Introduction

Worldwide, gastric cancer is the second common cause of cancer death, accounting for almost 10% of all cancer deaths [1]. Its incidence is estimated at 934,000 cases, among which 41% are in China [2]. Most patients presented with metastatic or unresectable disease at the time of diagnosis [3]. For these surgically unfit patients, palliative chemotherapy is the main choice of treatment. However, no globally accepted standard of first-line regimen is defined, while a platinum-fluoropyrimidine doublet, with or without epirubicin or docetaxel, is most commonly used [4–7]. As a new generation chemotherapy drug, docetaxel, which is a semisynthetic taxane, promoting the assembly and stabilization of microtubules to inhibit the depolymerization [8], has been used more and more extensively with potent effects [9–11]. Nevertheless, resistance to docetaxel did occur in several kinds of tumours [12,13]. Thus, identification of suitable biomarkers for predicting chemosensitivity to docetaxel may be a key role for improving therapeutic effects for patients, as well as those with advanced gastric cancer.

Forkhead box protein M1 (FOXM1), characterized by a 100 amino acid winged-helix DNA binding domain, is a newly unified family member of Forkhead transcription factor [14]. Previous researches indicated that FOXM1, activated by the Ras-mitogen-activated protein kinase and hedgehog signalling pathway [15,16] played an important role in cell cycle by promoting both the transition from G1 to S phase and the progression to mitosis through genes of Cdc25B, cyclin-dependent kinases1 and p27^KIP^ [17,18]. Recent studies have demonstrated that FOXM1 overexpressed in gastric cancer and that elevated FOXM1 promoted tumour development in various kinds of cancers, correlated closely with poor outcome [13,19–21]. In addition, in current research, FOXM1 amplification was reported to confer acquired paclitaxel resistance in breast cancer by interfering with the process of microtubule polymerization, showing implications in resistance to chemotherapy strongly [22]. Given the overlapping roles of docetaxel and FOXM1 upon affecting the microtubule dynamics of mitosis progression in tumour cells, it is intriguing to suggest that FOXM1 may correlate with docetaxel resistance through altering the microtubule dynamics and even regulating microtubule-associated protein such as Stathmin and mitotic centromere–associated kinesin (MCAK) in gastric cancer cells, about which little research has been carried out.

In this study, we intend to investigate the correlation between FOXM1 expression and chemotherapy response to docetaxel in gastric cancer cells and further explore its possible mechanism, trying to provide a support to chemotherapy choice for gastric cancer patients in clinical practice.

## Materials and methods

### Cell lines and culture conditions

Three gastric cancer cell lines, SGC-7901, AGS and MKN-28, which have different expression levels of FOXM1, were obtained from the central laboratory of Affiliated Hospital of Qingdao University Medical College. All the three cell lines were cultured in RPMI 1640 (Thermo scientific, Beijing, China), supplemented with 10% FBS (Thermo scientific) and 1% Penicillin/Streptomycin, and incubated in 5% CO_2_ at 37°C. The semisynthetic taxane, docetaxel (Selleckchem, Houston, TX, USA), was dissolved in dimethyl sulfoxide (DMSO) and diluted to a final concentration of 0.015, 0.020 and 0.15 mg/l, whereas thiazole antibiotic thiostrepton (BBI, Boston, MA, USA), which has been previously shown to inhibit FOXM1 expression, was prepared to be a solution at the concentration of 16 mg/l with DMSO before use [23].

### MTT assay

For MTT assays, 5000 cells were seeded overnight in 96-well plates and then cultured in 100 μl of fresh medium that contained various concentrations of docetaxel for 48 hrs. Each concentration was repeated in triplicate. After that, MTT solution (20 μl, 5 mg/ml in PBS) was added to each well and the plate was incubated for 4 hrs at 37°C. The solution was removed and 200 μl of DMSO was added to each well. After 10 min. of vibration mixing, the optical density at 490 nm was measured using a microplate reader (Bio-Rad, Hercules, CA, USA).

### Western blot analysis

Whole-cell lysates were prepared from gastric cancer cell lines, which were in logarithmic growth phase or at indicated periods of time. Total proteins were fractionated using sodium dodecyl sulphate polyacrylamide gel electrophoresis and transferred onto Polyvinylidene fluoride membrane. Anti-FOXM1 (dilution 1:1000; Epitomics, Burlingame, CA, USA), anti-Stathmin (1:1000; Epitomics), anti-MCAK (1:500; Abcam, Cambridge, UK) and anti-GAPDH (dilution 1:2000; CWBIO, Beijing, China) polyclonal antibodies were used as primary antibodies. For tubulin fractionation, α-tubulin antibody (1:1000; Santa, Santa Cruz, CA, USA) and β-tubulin antibody (1:1000; Santa) were used for analysis. The signals were detected using the VILBER enhanced chemiluminescence system (Vilber Lourmat, Marne-la-Vallée, France) according to the manufacturer*s instructions.

### Plasmids and transfections

The human FOXM1 expression vector pcDNA3.1-FOXM1, pcDNA3.1-Stathmin, siRNA-FOXM1, siRNA-Stathmin, siRNA-MCAK were obtained from the centre library of Affiliated Hospital of Qingdao University Medical College. For transfections, cells were seeded to a 40–50% confluence state and transfected with pcDNA3.1-FOXM1, pcDNA3.1-Stathmin, siRNA-FOXM1, siRNA-Stathmin, siRNA-MCAK, pcDNA3.1 or non-specific siRNA with Lipofectamine 2000 agent (Invitrogen, Carlsbad, CA, USA) in accordance with the manufacturer*s protocol. For the human Stathmin promoter plasmids containing firefly luciferase reporters, an internal control pMiniTK-RL, which included a full-length Renilla luciferase gene under the control of a minimal thymidine kinase promoter, was cotransfected into gastric tumour cell lines in triplicate. Twenty-four hours after transfection, cells were harvested for Luciferase assay.

### Molecular evolution assay

The gastric cancer cell line AGS was treated with 0.015 mg/l docetaxel for 72 hrs when cells reached a confluency of 80%. After treatment, docetaxel-containing medium was replaced by fresh medium. As soon as cells were recovered, they were seeded for RNA isolation, cell lysis (protein), MTT assays and the next treatment cycle. In this manner, several rounds of molecular evolution assay were performed. To obtain the appropriate rate of cell death in the molecular evolution assay, several docetaxel concentrations were tested in a preliminary experiment. Thereby, 0.015 mg/l was obtained as the most suitable concentration.

### Semi-quantitative RT-PCR

Total cellular RNA was extracted from cell pellets of each cell lines with Trizol reagent. The amount of RNA was determined by BJKO assay system (BJKO, Beijing, China) and part of them was reverse transcribed using the Reverse Transcription System (TaKaRa, Dalian, China). The primer sequences for PCR amplification were as follows: FOXM1 sense 5′-TAT TCA CAG CAT CAT CAC AGC A-3′ and antisense 5′-GAA GGC TCC TCA ACC TTA ACC T-3′; Stathmin sense 5′-GCC AGT GTC CTT TAC TTT CCC TCC-3′ and antisense 5′-TTC AGT TTC TCC CCT TAG GCC C-3′; MCAK sense 5′-GAT GGA AGC CTG CTC TAA CG-3′ and antisense 5′-GAG CAG ATT CCG CTT TGT TC-3′; GAPDH sense 5′-ACC ACA GTC CTG CAT GCC AC-3′ and antisense 5′-TCC ACC ACC CTG TTG CTG TA-3′. To ensure experiment accuracy, all reactions were performed in triplicate. The integrity of all RNA samples was verified by RT-PCR for GAPDH in each sample through gel imaging system (Vilber Lourmat). The value of FOXM1 expression was divided by that of GAPDH in each sample.

### Tubulin assay

Separation of polymerized and soluble fractions was carried out in accordance with previously published assays [22]. Cells were seeded at 80% confluency in 24-well plates. The following day, they were treated with 0 or 0.020 mg/l docetaxel for 48 hrs. Cells were collected in hypotonic buffer (1 mM MgCl_2_, 2 mM EGTA, 0.5% Nonidet P-40, 20 mM Tris-HCl pH 6.8) and centrifuged for 10 min. at room temperature (14,000 rpm). The supernatant was used as the soluble fraction, while the pellet made up the polymerized fraction. Samples were analysed by western blot.

### Luciferase reporter assay

For promoter analysis, 24 hrs after transfection, cells were then collected, washed twice in PBS and harvested for firefly/Renilla luciferase assays using the Dual-Glo™ Luciferase reporter assay system (Promega, Madison, WI, USA) according to the manufacturer*s instructions. After that, luminescence was read using the TECAN Safire II (Tecan, Männedorf, Swiss) plate reader.

### Chromatin immunoprecipitation (ChIP)

Chromatin immunoprecipitation assays were performed with the ChIP assay kit (Cell Signaling, Boston, MA, USA). The resulting precipitated DNA samples were analysed by semi-quantitative RT-PCR. The following primers were used for PCR: Stathmin sense 5′-CAA ATG TGC TTG CCT TTT AGC C-3′ and antisense 5′-TGG GAT TAC AGA TGT GAG CCA CC-3′ for −5397, and Stathmin sense 5′-CAC GGT CAG ACC AAT TTC T-3′ and antisense 5′-TGA TAG GGG AGG AAG AGC AA-3′ as a non-specific control. The PCR products were resolved electrophoretically on a 2% agarose gel and visualized by ethidium bromide staining.

### Patient tissue specimens

Our study included 103 patients who underwent gastrectomy and D2 lymphadenectomy at the Affiliated Hospital of Qingdao University Medical College from January 2007 to November 2007. All patients met the criteria: (*i*) Tumours were confirmed to be gastric adenocarcinoma histologically. (*ii*) None had received pre-operative treatment such as chemotherapy or radiotherapy. (*iii*) Everyone was available of a 5-year retrospective follow-up data. The study was approved by the ethical committee of our institute. Clinic-pathological information obtained from patient*s operative and pathological reports was summarized in the Tables.

### Immunohistochemical analysis

An immunohistochemical analysis of human gastric cancer specimens was performed with antibody against human FOXM1 (dilution 1:100; Epitomics) or Stathmin (dilution 1:200; Epitomics). In every case, negative control reaction was set with PBS replacing FOXM1 or Stathmin antibody, while the known positive-stained section was used as positive control. We quantitatively scored tissue sections according to the percentage of positive cells and staining intensity, which has been described previously [19].

## Results

### Elevated FOXM1 expression correlates with resistance to docetaxel

**Fig. 1:**
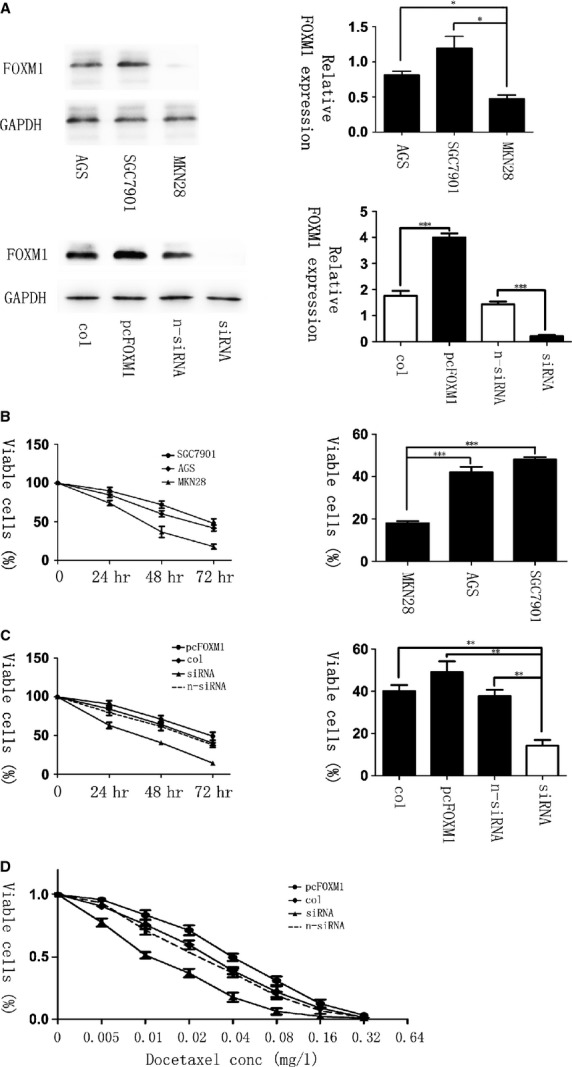
Elevated levels of forkhead box protein M1 (FOXM1) correlate with resistance to docetaxel in gastric cancer. (**A**, Top) The expression of FOXM1 in three gastric cancer cell lines: AGS, SGC-7901 and MKN-28, shown by western blot. (Bottom) The expression of FOXM1 after transfected with pcDNA3. 1, pcDNA3. 1-FOXM1, non-specific siRNA or FOXM1-siRNA in gastric cell lines AGS, analysed by western blot 48 hrs later. (**B**) AGS, SGC-7901 and MKN-28 cells were treated with 0.02 mg/l of docetaxel for 0, 24, 48 and 72 hrs. MTT assay was performed to test the cell viability. (**C**) Gastric cell lines AGS were treated with docetaxel at the concentration of 0.02 mg/l after FOXM1-siRNA or pcDNA3, 1-FOXM1 transfection for 72 hrs. Cell growth curves were drawn by MTT assays. The IC50 in FOXM1 knockdown, overexpressed, non-specific siRNA and pcDNA3, 1 transfected groups was 0.012, 0.040, 0.024 and 0.027 mg/l respectively (**D**). *P ≤ 0.05; **P ≤ 0.01; ***P ≤ 0.001 significant.

We initiated our study by investigating whether there is an association between the expression of FOXM1 and chemotherapy response to docetaxel. To explore it, the human malignant gastric cell lines SGC-7901, AGS and MKN-28, which has different expression levels of FOXM1 (Fig. 1A), were treated with 0.02 mg/l of docetaxel for 72 hrs. With the detection of MTT assay, only 15–20% of the MKN-28 cells survived after treatment, whereas the survival in FOXM1-overexpressed cells was greater than 40% (*P* < 0.001, Fig. 1B), indicating that the expression of FOXM1 correlated with docetaxel therapeutic efficacy significantly. To further confirm this result, we then transfected pcDNA3.1-FOXM1 and FOXM1-siRNA into AGS cell lines (Fig. 1A) and incubated them at the same drug concentration for 3 days. As shown by cell growth curve, the cell viability was absolutely lower in samples with FOXM1 knockdown, whereas the pcDNA3.1-FOXM1–transfected cells had higher viable rate (*P* < 0.01, Fig. 1C). Moreover, the hypothesis that knockdown of FOXM1 in AGS sensitized the cells to docetaxel treatment was also demonstrated by IC50 calculations, 0.040 mg/l (pcDNA3.1-FOXM1), 0.027 mg/l (pcDNA3.1) and 0.024 mg/l (non-specific siRNA) *versus* 0.012 mg/l (siRNA FOXM1; Fig. 1D). These data indicated that FOXM1 can protect cells from docetaxol-induced cell damage.

### Molecular evolution of gastric cancer cells leads to a docetaxel-resistant phenotype and up-regulation of FOXM1

**Fig. 2:**
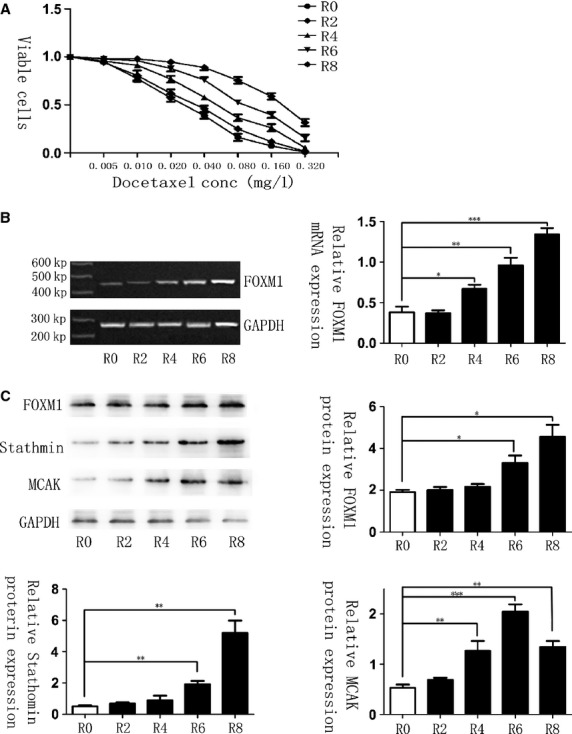
Docetaxel-resistant cell line shows elevated forkhead box protein M1 (FOXM1) mRNA and protein expression levels. (**A**) Cells after molecular evolution assay were treated with increasing concentrations of docetaxel, respectively, and their rates of cell viability were measured by MTT assay. R0 represents the untreated control cell line, whereas R2, R4, R6 and R8 represent AGS cells that are treated with 0.015 mg/l docetaxel for two, four, six and eight times, separately. IC50 for cells in R0, R2, R4, R6 and R8 was 0.026, 0.033, 0.054, 0.098, 0.190 mg/l. (**B**) FOXM1 mRNA transcript levels in R0, R2, R4, R6 and R8 cells were determined by RT-PCR analysis. (**C**) Western blot analysis was performed to detect the relative protein expression levels of FOXM1, Stathmin and mitotic centromere–associated kinesin in the different treated round. Results shown were derived from at least three independent experiments. Statistical analysis was performed with Student*s *t*-tests. *P ≤ 0.05; **P ≤ 0.01; ***P ≤ 0.001 significant.

To confirm that chemoresistance can also lead to the up-regulation of FOXM1, we established the molecular evolution assay, where the malignant human gastric cell line AGS was treated with docetaxel for several cycles. After each treatment round, cells were harvested for MTT assay, as well as RNA and protein isolation to investigate chemosensitivity changes and gene expressions. As a result, MTT assays revealed that cells in sequential treatment cycles had increasing IC50 calculations (Fig. 2A), demonstrating that the resistance to docetaxel rose especially from the fourth treatment cycle on. In addition, changes in the levels of FOXM1 could be observed simultaneously. PCR result showed that the level of FOXM1 was up-regulated after the fourth treated round (*P* < 0.05, Fig. 2B), while the expression of FOXM1 altered correspondingly with mRNA levels (*P* < 0.05, Fig. 2C). Based on such treatment, AGS cells were finally succeeded to have a good tolerance of docetaxel to the concentration of 0.2 mg/l, which were regarded as the AGS-DOC^R^ cell lines. These results provided another aspect of evidence and fully proved that FOXM1 could mediate the therapeutic resistance to docetaxel in gastric cancer.

### FOXM1 confers resistance to docetaxel by altering microtubule dynamics in preventing docetaxel-induced apoptosis

Several mechanisms to combat palitaxol-induced apoptosis have been reported. Namely, up-regulation of MDR1 (multi-drug resistant protein 1), a P-Glycoprotein family member can shuttle toxins out of cells; up-regulation of the CIAP (inhibitors of apoptosis) family members including Survivin; and the altered microtubule dynamics [24]. Considering that docetaxel is a member of microtubule-stabilizing agent family, similar to palitaxel, and FOXM1 also participated in the progression of mitosis, we suggested that hypothesis that altered microtubule dynamics mediated by FOXM1 could prevent docetaxol-induced apoptosis, which caused docetaxel resistance in gastric cancers.

**Fig. 3:**
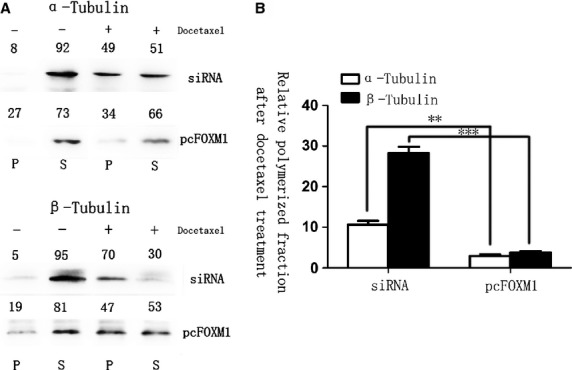
Forkhead box protein M1 (FOXM1) alters the microtubule dynamics in preventing docetaxel-induced apoptosis. Polymerized and soluble tubulin fractions from docetaxel untreated and treated FOXM1-siRNA and pcDNA3, 1-FOXM1–transfected cell lines were generated by centrifugation. Western blot was used to assay α-tubulin and β-tubulin ratios in polymerized and soluble fractions. Relative percentages are shown above western blot (Left). The soluble-to-polymerized microtubule fractions after docetaxel treatment were significantly inhibited in FOXM1-overexpressed group, analysed by *t*-test both for α-tubulin and for β-tubulin (Right). **P ≤ 0.01; ***P ≤ 0.001 significant.

To examine its possibility, we compared the ratio of soluble to polymerized microtubule fractions after docetaxel treatment. Cell lysates were fractionated to obtain polymerized and soluble tubulin fractions in FOXM1-siRNA–transfected and FOXM1-overexpressed gastric cell lines that were left untreated or treated with docetaxol. Without drug treatment, cells showed similar tubulin ratios and most of the detectable tubulins were in the soluble form. Upon treatment with docetaxol, FOXM1 knockdown cells showed a dramatic shift towards the polymerized fraction. The FOXM1-expressing cells did show a shift towards the polymerized fraction, but the ratio was significantly lower (*P* < 0.01, Fig. 3). These data clearly indicated that through interfering in microtubule polymerization, the anti-tumour activity of docetaxel was inhibited by FOXM1 overexpression in gastric cancer cells, actually testifying our previous hypothesis.

### FOXM1 can promote docetaxel resistance through the microtubule-destabilizing protein Stathmin rather than MCAK in gastric cancers

**Fig. 4:**
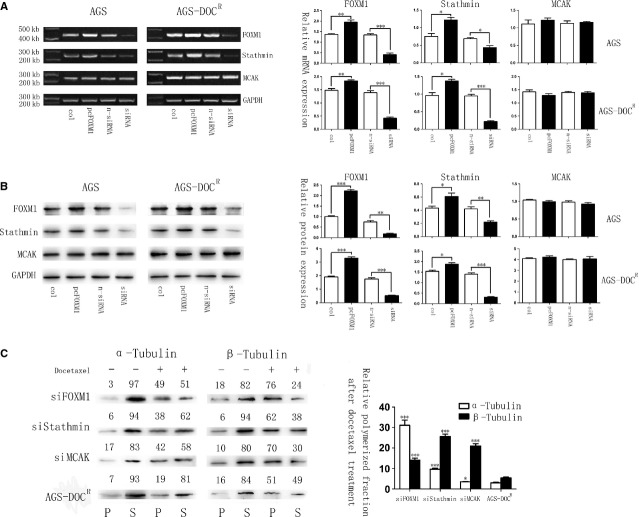
Forkhead box protein M1 (FOXM1) promotes docetaxel resistance through regulating the microtubule-destabilizing protein Stathmin rather than mitotic centromere–associated kinesin (MCAK) in gastric cancers. (**A**) mRNA levels of FOXM1, Stathmin and MCAK in AGS cells after transfected with pcDNA3. 1, pcDNA3. 1-FOXM1, non-specific siRNA or FOXM1-siRNA were determined by RT-PCR. (**B**) The protein expression levels of FOXM1, Stathmin and MCAK after transfections were shown by western blot analysis. (**C**) Polymerized and soluble tubulin fractions from siRNA -FOXM1, siRNA-Stathmin, siRNA-MCAK and non-specific siRNA-transfected AGS-DOC^R^ cell lines treated or untreated with docetaxel were detected by western blot (Left). The relative polymerized microtubule fractions of both α-tubulin and β-tubulin were significantly increased after the treatment of docetaxel in FOXM1, Stathmin and MCAK knockdown AGS-DOC^R^ cells (Right). *P ≤ 0.05; **P ≤ 0.01; ***P ≤ 0.001 significant.

It has been previously established that increased expression and activity of the microtubule-destabilizing protein Stathmin and MCAK can confer resistance to palitaxol-induced apoptosis in breast epithelial and CHO cell lines respectively [22,25]. The hallmark of increased drug resistance is a low ratio of soluble to polymerized tubulin [26], just as we observed in FOXM1-overexpressed cells. Therefore, we detected Stathmin and MCAK levels in gastric cells after each treatment round of molecular evolution assay and found that the expression levels of Stathmin and MCAK increased correspondingly with the resistance to docetaxel, demonstrating that Stathmin and MCAK expression correlated with docetaxel chemotherapy response significantly (Fig. 2C). Next, we examined whether Stathmin and MCAK are the downstream targets of FOXM1 in conferring docetaxel resistance in gastric cancer cells. Interestingly, the siRNA-FOXM1 and pcDNA3.1-FOXM1–mediated regulation of FOXM1 did not result in a corresponding alteration of MCAK in either the AGS or the AGS-DOC^R^ cell lines, whereas the level of Stathmin changed accordingly both at the protein and at the mRNA levels (Fig. 4A and B), indicating that FOXM1 is not the sole regulator of MCAK in these cells. The requirement of FOXM1, Stathmin and MCAK expression for AGS-DOC^R^ docetaxel resistance was further examined using siRNA-mediated knockdown of these genes. The knockdown of FOXM1, Stathmin and MCAK in AGS-DOC^R^ cells significantly increased the polymerized fraction of microtubule after the treatment with docetaxel (Fig. 4C). Moreover, following the silence of FOXM1, an increase in polymerized fraction rate was observed with the down-regulation of Stathmin, whereas the expression levels of MCAK were still maintained (Fig. 4A and B), suggesting that Stathmin is the downstream target of FOXM1 that overcomes gastric cell apoptosis induced by docetaxel.

### FOXM1 regulates Stathmin expression at the promoter level in gastric cancers

**Fig. 5:**
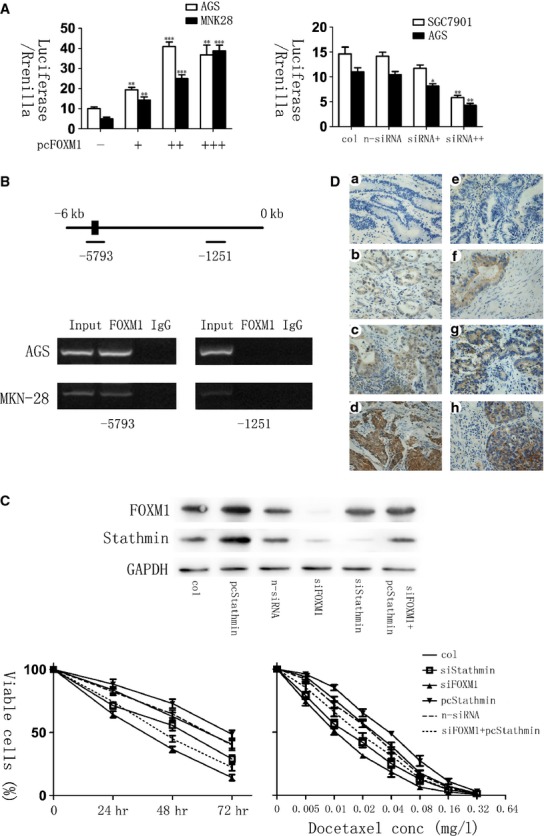
Forkhead box protein M1 (FOXM1) correlated with Stathmin in gastric cancers. (**A**) The luciferase activity of Stathmin promoter–reporter vectors was modulated by FOXM1 levels in human gastric cell lines. (Left) MNK-28 and AGS cells were transfected with Stathmin promoter–reporter vectors and increasing amounts (0.2, 0.4, 0.6 ng) of pcDNA3, 1-FOXM1. (Right) AGS and SGC-7901 cells were transfected with Stathmin promoter–reporter vectors and transfection reagents, non-specific siRNA or different amount (10 and 20 nM) of FOXM1-siRNA. pcDNA3,1 was used as a control. The relative Stathmin promoter activities were measured 24 hrs after transfection, and the activities in the treated groups were expressed as the fold or percentage of that in their respective control groups. (**B**) Chromatin immunoprecipitation assay (ChIP) was performed in AGS and MKN-28 cells using an antibody specific to FOXM1 or IgG as a control. PCR was used to amplify the region surrounding the putative FOXM1 binding site at −5793 upstream of the transcriptional start site and the region surrounding −1251 as a non-specific control. Representative PCR results are shown. (**C**) Silencing FOXM1 sensitized docetaxel-resistant gastric cancer cells, and Stathmin overexpression rescued chemoresistance in FOXM1-silenced AGS-DOC^R^ cells. Western blot analysis reveals the expression of genes in AGS-DOC^R^ cells expressing pcDNA3,1-Stathmin, si-FOXM1, si-Stathmin or si-FOXM1 plus Stathmin (Top). MTT assay shows cell survival in AGS-DOC^R^ cells expressing pcDNA3,1, pcDNA3,1-Stathmin, non-specific siRNA, si-FOXM1, si-Stathmin or si-FOXM1 plus Stathmin treated with docetaxel and the IC50 was 0.026, 0.036, 0.024, 0.011, 0.014, 0.019 mg/l respectively (Bottom). (**D**) Immunohistochemical staining for FOXM1 and Stathmin antibody in human gastric cancer tissues (×400). The paraffin-embedded gastric tissues were stained with antibodies FOXM1 (A, B, C, D) and Stathmin (E, F, G, H). *P ≤ 0.05; **P ≤ 0.01; ***P ≤ 0.001 significantly.

To investigate whether Stathmin is a downstream signalling target of FOXM1 in gastric cancer, we next cotransfected Stathmin promoter–reporter plasmids with FOXM1-overexpressed vectors or FOXM1-siRNA into malignant human gastric cell lines. The results of luciferase reporter assays were shown in Figure 5A, which suggested that the relative luciferase activity was elevated generally with the increased concentration of pcDNA3,1-FOXM1 in AGS and MKN-28 cells, while FOXM1-siRNA inhibited the luciferase activity driven by the Stathmin promoter in AGS and SGC-7901 cells, demonstrating that FOXM1 is involved in the transactivation of Stathmin through the promoter region. In addition, ChIP with an FOXM1 antibody showed that the enrichment of the Stathmin promoter region was higher in AGS than MKN-28 cells (Fig. 5B), indicating that the RNA and subsequent protein increase in Stathmin in FOXM1 expressing lines is likely as a result of a direct interaction of FOXM1 with the Stathmin gene promoter. Overall, these findings verified that FOXM1 directly targeted and up-regulated the microtubule-destabilizing protein Stathmin, and then prevented the tubulin polymerization, eventually mediated the resistance to docetaxol-induced apoptosis in gastric cancer cells.

### Stathmin mediates docetaxel resistance in FOXM1-silenced gastric cancer cells

To determine whether docetaxel resistance in gastric cancer cells is dependent on Stathmin, we inhibited Stathmin expression by Stathmin-siRNA in AGS cells. Stathmin knockdown significantly decreased cell viability after the treatment of docetaxel for 3 days and obviously increased the chemosensitivity to docetaxel in AGS cells (Fig. 5C). Moreover, after silencing the expression of FOXM1, we further analysed the cell response to docetaxel with the transfection of pc3, 1DNA- Stathmin in those previously Stathmin–down-regulated cells. Conversely, Stathmin re-expression could partially attenuate docetaxel-induced cell apoptosis in FOXM1-knockdown gastric cancer cells and decrease their sensitivity to docetaxel as a result of MTT assay (Fig. 5C). What is more, it can be simultaneously observed that the expression level of FOXM1 was elevated in FOXM1-knockdown cells with Stathmin transfection compared with those without, prompting that the up-regulation of Stathmin may be related to the increase in FOXM1 somehow. In any case, these data generally suggested that the expression of Stathmin could mediate docetaxel resistance in gastric cancer cells after the silence of FOXM1.

### FOXM1 and Stathmin expression levels were correlated in human gastric cancer specimens and FOXM1 was independently predictive of poor prognosis

The expression of FOXM1 and Stathmin in human gastric cancer samples was analysed by immunohistochemical staining. FOXM1 and Stathmin showed overlapping expression in gastric cancer cells, among which, the expression of FOXM1 is located in the nucleus and cytoplasm of cells, while Stathmin protein mainly expressed in the cytoplasm (Fig. 5D). Furthermore, we performed an immunohistochemical analysis of FOXM1 and Stathmin proteins in 103 gastric cancer samples. As determined by Pearson*s correlation test, FOXM1 and Stathmin expression levels were positively correlated in those gastric cancer samples (*P* = 0.029, Table 1). Moreover, through cox proportional hazard model, FOXM1 expression levels were significantly identified as an independent prognostic factor for survival duration in gastric cancer patients (*P* < 0.01, Table 2). Nevertheless, although Stathmin expression was shown to be associated with poor survival by univariate analysis (*P* < 0.01), it was not independently correlated with the prognosis in gastric cancer patients as a result of multivariate analysis (*P* > 0.05, Table 2).

**Table 1:** Association between FOXM1/Stathmin expression and clinic-pathological factors in 103 patients after gastrectomy

Viable	Entire group (*n* = 103)	FOXM1 negative	FOXM1 positive	*P*
Age				
≤50 years	17	4	13	1
>50 years	86	18	68	
Gender				
Male	68	15	53	0.809
Female	35	7	28	
Size				
≤5 cm	73	15	58	0.754
>5 cm	30	7	23	
Depth of tumour invasion				
T1–2	40	7	33	0.446
T3–4	63	15	48	
Lymph node metastasis				
Negative	40	8	32	0.789
Positive	63	14	49	
Degree of differentiation				
Undifferentiated	75	17	58	0.596
Differentiated	28	5	23	
Venous invasion				
Negative	61	14	47	0.701
Positive	42	8	34	
Neural invasion				
Negative	56	10	46	0.344
Positive	47	12	35	
Bomann histological classifications (*n* = 77)				
Bomann I	4	1	3	0.436
Bomann II–III	66	14	52	
Bomann IV	7	3	4	
TNM staging				
I–II	45	7	38	0.206
III–IV	58	15	43	
Stathmin status				
Negative	49	15	34	0.029*
Positive	54	7	47	

* *P* < 0.05.

**Table 2:** Results of univariate and multivariate analysis of survival in 103 gastric cancer patients by Cox proportional hazard model

		Univariate analysis			Multivariate analysis		
			
Viable	*n*	OR	95.0% CI	*P*	OR	95.0% CI	*P*
Age (≤50 years or >50 years)	19/84	0.738	0.363–1.499	0.400			
Gender (F/M)	68/35	0.759	0.440–1.308	0.320			
Size of tumour (≤1 cm/>1 cm)	10/93	0.287	0.17–0.483	0.001**	0.507	0.285–0.901	0.021*
Depth of tumour invasion (T1-2/T3-4)	40/63	0.209	0.108–0.404	0.002**	10.798	1.993–14.561	0.009**
Lymph node metastasis (negative/positive)	40/63	0.179	0.09–0.355	0.002**	0.47	0.197–1.121	0.089
Degree of differentiation (undifferentiated/differentiated)	75/28	1.041	0.593–1.825	0.889			
Venous invasion (negative/positive)	61/42	0.375	0.225–0.628	0.001**	0.732	0.414–1.294	0.283
Neural invasion (negative/positive)	56/47	0.917	0.553–1.523	0.739			
FOXM1 expression (negative/positive)	22/81	2.277	1.081–4.796	0.030*	6.123	2.645–14.173	0.001**
TNM staging (I–II/III–IV)	45/58	0.118	0.059–0.237	<0.001**	0.008	0.001–0.087	<0.001**
Stathmin status (negative/positive)	49/54	0.166	0.090–0.307	<0.001**	1.491	0.491–4.528	0.481

* *P* < 0.05;

** *P* < 0.01.

### Thiostrepton can overcome docetaxel resistance in gastric cancer cells through down-regulation of FOXM1

**Fig. 6:**
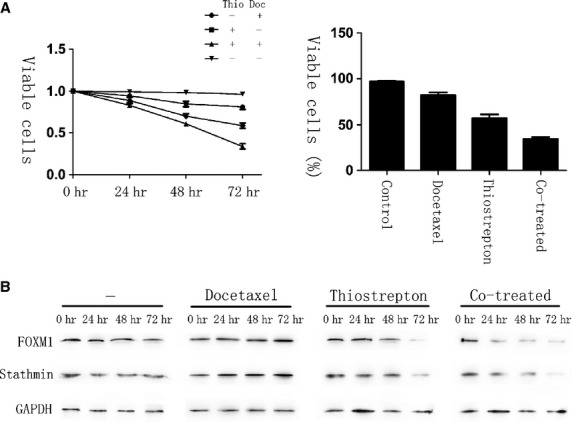
Thiostrepton can reverse docetaxel resistance in gastric cancer cells. AGS-DOC^R^ cells were treated with dimethyl sulfoxide (vehicle control), 0.15 mg/l docetaxel, 16 mg/l thiostrepton or a combination of 0.15 mg/l docetaxel and 16 mg/l thiostrepton for 72 hrs. (**A**) The percentage of viable cells in different treatment group is shown at each time-point by MTT assay. (**B**) Cell lysates were prepared at 0, 24, 48 and 72 hrs after treatment, and the expression of forkhead box protein M1, Stathmin and GAPDH were analysed by western blot analysis.

To test if FOXM1 inactivation is a viable strategy for overcoming docetaxel resistance, we studied the effects of AGS-DOC^R^ cells treated with the thiazole antibiotic thiostrepton, which is a proteasome inhibitor that can suppress FOXM1 expressions [27]. In result, MTT assays revealed that docetaxel-resistant cells exhibited a significant reduction in the rate of cell viability after treated with thiostrepton or in combination with docetaxel, whereas the viable rate was observed significantly higher after the single treatment of docetaxel. Moreover, 72 hrs later, drug-resistant cells treated with thiostrepton alone showed a 59% cell survival rate, whereas the combination of docetaxel and thiostrepton indicated a synergy, exhibiting a cell survival rate of 34% in this experiment (Fig. 6A).

In the drug-resistant cells co-treated with thiostrepton and docetaxel, the down-regulation of FOXM1 and its downstream target Stathmin were observed more evidently compared with incubating by either of them alone, whose occurrence was also earlier (Fig. 6B). The larger reduction and shorter time needed for FOXM1 and Stathmin down-regulation in the co-treated cells may reflect the higher levels of cell death happened when both drugs were administered together, which proved that a synergy did exist between the two drugs. Taken together, shown by our research, the resistance of docetaxel is able to be reversed in gastric cancer cells through the inhibition of FOXM1.

## Discussion

In this study, we demonstrated that the expression of FOXM1 mediated the resistance to docetaxel in gastric cancer cells, for which elevated levels of FOXM1 was shown to correlate with lower drug susceptibility, whereas the molecular evolution assay of AGS resulted in significantly more resistant cells possessed FOXM1 overexpression. To further investigate the mechanism, we found that FOXM1 performed a crucial role in docetaxel-resistant gastric cancer cells by directly targeting and up-regulating the microtubule-destabilizing protein Stathmin, which partially rescued FOXM1-knockdown inhibitory effect on docetaxel resistance, and then inhibiting the polymerization of tubulin. Moreover, after treating cells with FOXM1 inhibitor thiostrepton, the acquired drug resistance was reversed with the down-regulation of FOXM1, and thus, the inactivation of FOXM1 is essential for reversing docetaxel resistance, and targeting FOXM1 could potentially be a better therapeutic strategy for overcoming the resistance to docetaxel.

In gastric cancer treatment, classical chemotherapy is largely used besides surgery operation, radiotherapy and novel-targeted therapy approaches. For instance, docetaxel is commonly used as single agent or in combination with other drugs like platinum and fluoropyrimidine (DCF regimen) in a neo-adjuvant or advanced stage setting [6,7]. The chemotherapy based on docetaxel may be effective, because docetaxel was reported to lack cross-resistance with other anti-tumour drugs [9]. However, the resistance to docetaxel did occur and FOXM1 was shown to be a critical molecule, but the mechanism remained unclear [13]. Currently, on the basis of the overlapping role of FOXM1 and docetaxel in the progression of mitosis, we provided hypothesis that FOXM1 may prevent docetaxol-induced apoptosis through altering the dynamics of microtubule and further examined the possibility of it [17,18]. As a result, not only do we demonstrate that microtubules in FOXM1-overexpressed cell lines fail to polymerize in response to docetaxol treatment but also that FOXM1 directly targeted and up-regulated the microtubule-destabilizing protein Stathmin rather than MCAK, which also correlated with docetaxel resistance in gastric cancer cells, indicating that FOXM1 could bound at the Stathmin gene promoter and prevented the tubulin polymerization to induce docetaxel resistance in gastric cancers. To further confirm that association, we detected the expression levels of FOXM1 and Stathmin in gastric cancer tissues. Consequently, our immunohistochemical analyses revealed that FOXM1 expression levels were significantly correlated with Stathmin levels in gastric cancer specimens and both of them were associated with the poor survival as a result of univariate analysis, while only the expression of FOXM1 was identified as an independent prognostic marker for survival duration in post-operational gastric cancer patients, which provided clinical evidence to our mechanism researches.

As a down-stream target of FOXM1, Stathmin is the founding member of microtubule-destabilizing proteins family that have a critical role in the regulation of mitosis [28]. Numerous studies have noted that the expression of Stathmin was up-regulated in several types of cancer and that overexpressed Stathmin protects cancer cells from apoptosis and enhances chemotherapy resistance by promoting microtubule depolymerizing [29–32]. However, the mechanism of Stathmin up-regulation in gastric cancer is still unknown. In this study, we found that FOXM1 crucially regulated Stathmin expression by directly interacting with the promoter through FOXM1-binding site. Furthermore, when Stathmin was overexpressed in FOXM1-knockdown gastric cancer cells, they became resistant to docetaxel, indicating that the FOXM1–Stathmin axis plays a critical role in docetaxel resistance. Moreover, compared with those FOXM1-knockdown cells, the transfection of pcDNA3.1-Stathmin was found to be able to slightly elevate the expression levels of FOXM1 in gastric cancers, in addition that the FOXM1 protein level is also increased after pcSathmin transfected. These findings suggested that a feedback loop may exist between the two genes and about which more investigations are required. However, only a partial rescue effect *in vitro* was shown by Stathmin overexpression, implying that some other FOXM1 target genes may be involved in docetaxel resistance. Accordingly, as a microtubule motor protein that is also known as Kif2c, MCAK has been revealed to mediate paclitaxel resistance in previous studies and thus analysed by our research [25]. Interestingly, although MCAK levels are elevated in the AGS-DOC^R^ and pcDNA3.1-FOXM1–transfected cells, evidence suggested that FOXM1 is not the sole regulator of MCAK. For instance, MCAK expression was not changed correspondingly with the regulation of FOXM1 levels. In addition, when we transfected siRNA-FOXM1 into AGS-DOC^R^ cells, the expression of MCAK still maintained. Furthermore, although the expression levels of MCAK were higher in the AGS-DOC^R^ cells compared with the parental cells, transient transfection of AGS with pcDNA3.1-FOXM1 failed to up-regulate MCAK expression. All the supplementary evidence implies that additional regulators are needed, although FOXM1 plays a part in their activation.

It has been mentioned that FOXM1 was overexpressed in about 68–78% of gastric cancers [13,33]. For these FOXM1-elevated patients, docetaxel may not be the best choice among the various chemotherapeutic agents. In this case, our study may give implications in the development of a new treatment regime, suggesting that the target gene of FOXM1 would be more efficient for docetaxel-resistance patients. For example, Thiostrepton, a proteasome inhibitor that can suppress FOXM1 expression, was shown to have a synergy with docetaxel for causing a substantial increase in the amount of docetaxel-induced cell deaths and could reverse the acquired docetaxel resistance in gastric cancer cells by our research. In addition, inhibition of FOXM1 function by a cell-penetrating ARF (26–44) peptide has also been revealed to lead to reduced tumour cell proliferation and angiogenesis in hepatocellular carcinoma [34], exhibiting the potential of inhibitors for FOXM1 in becoming new anticancer therapeutics. Moreover, revealed as a down-stream target of FOXM1 in our current research, the reversal of Stathmin-mediated microtubule destabilization has been previously indicated as a novel synergistic therapeutic intervention with a low dose of anti-microtubule agents [32]. When breast cancer xenografts were treated with low doses of anti-Stathmin therapy and palitaxol, a dose-dependent inhibition of proliferation and clonogenicity was markedly enhanced, and a regression was shown in a majority of tumours, while some tumours stopped growing completely [35], demonstrating a great value of anti-Stathmin therapy in the treatment of docetaxel-resistant gastric cancer patients. Through these investigation of mechanism in docetaxel-resistant gastric cancers, we can conclude that therapies aimed at reducing FOXM1 or its down-stream target Stathmin will serve as a method of sensitizing tumour cells to docetaxel therapies, and with the addition of a FOXM1 or Stathmin inhibitor to a chemotherapeutic regimen, the drug effective doses would be lower and the side effects for patients could be potentially reduced.

Although our study gave the evidence to the hypnosis that FOXM1 mediates resistance to docetaxel in gastric cancer and expounded the mechanism, the accurate binding sites at gene level were not revealed yet. Moreover, considering that FOXM1 was involved in various aspects of physiological progression of tumourigenesis, some other mechanism such as regulating the expression of DNA-repaired genes may also correlate with the occurrence of docetaxel resistance, which need further explorations.

## Conclusion

In summary, FOXM1 is a critical mediator of docetaxel sensitivity and resistance in gastric cancer cells. Therefore, FOXM1 can be a useful marker for predicting and monitoring docetaxel response. Through the inhibition of FOXM1, it is possible that docetaxel resistance can be reversed, and FOXM1 might be a new therapeutic target in docetaxel-resistant gastric cancer.
